# Prognostic value of existing scoring systems for early mortality in phase I immunotherapy trials: a head-to-head real-world cohort study

**DOI:** 10.3389/fonc.2026.1849425

**Published:** 2026-07-20

**Authors:** Paloma Sangro, Ana Landa-Magdalena, Miguel Sogbe, Teresa Zumarraga, Ignacio Matos, Mariano Ponz-Sarvise, Marina Crespo, María Esperanza Rodriguez-Ruiz, Anna Vilalta-Lacarra, Eduardo Castañón

**Affiliations:** 1 Liver Unit and HPB Oncology Area, Cancer Center Clínica Universidad de Navarra (CCUN), Madrid, Spain; 2 Centro de Investigación Biomédica en Red en el Área temática de Enfermedades Hepáticas (CIBERHED), Pamplona, Spain; 3 Instituto de Investigación Sanitaria de Navarra (IDISNA), Pamplona, Spain; 4 Phase I Clinical Trial Unit for Oncology. Oncology Department. CCUN, Pamplona, Spain; 5 Liver Unit and HPB Oncology Area, CCUN, Pamplona, Spain; 6 Phase I Clinical Trial Unit for Oncology. Oncology Department. CCUN, Madrid, Spain; 7 Unidad de Gestión Clínica de Oncología de Gipuzkoa, San Sebastián, Spain

**Keywords:** ethical bias, immunotherapy, Ockham’s razor, phase I clinical trials, prognostic scores

## Abstract

**Background:**

Patients enrolled in phase I oncology clinical trials are expected to survive more than 3 months, yet up to 15% may die before this threshold. Several prognostic scores have been developed to identify patients unlikely to derive benefit from trial participation. We aimed to compare the prognostic performance of five described scores.

**Methods:**

This retrospective study included 844 patients treated with immunotherapy in phase I trials at Clínica Universidad de Navarra (2016-2023). Prognostic scores (RMH, GRIm, LIPI, PIPO, ROPRO) were calculated according to their original definitions. Discriminative performance was assessed using Harrell’s C-index, 3-month AUC, and time-dependent AUC.

**Results:**

Median OS was 9.08 months. All scores demonstrated statistically significant prognostic stratification. C-index values were: RMH 0.58, GRIm 0.55, LIPI 0.66, PIPO 0.63, and ROPRO 0.69. AUC at 3 months was highest for ROPRO (0.76). However, no score identified a subgroup with a median OS below 3 months.

**Conclusions:**

All five scores stratified prognosis but failed to select patients with OS below 3 months. These findings challenge their use as rigid exclusion criteria and support their role as stratification factors. New models integrating dynamic clinical or molecular biomarkers are needed to improve patient selection in phase I immunotherapy trials.

## Background

Phase I clinical trials (CTs) are the entry point for evaluating novel oncologic therapies primarily designed to assess safety, determine optimal dosing, and characterize the pharmacokinetic and pharmacodynamic profiles of investigational agents (1). Historically, these studies enrolled heavily pretreated patients with advanced malignancies and limited therapeutic alternatives, for whom participation offered a last-resort opportunity for clinical benefit. However, the landscape of early-phase oncology research has evolved substantially over the past decade. The advent of molecularly targeted agents and immune checkpoint inhibitors (ICIs) has progressively shifted phase I trial enrollment toward earlier lines of therapy, increasingly including patients with better performance status and more favorable baseline prognosis. This paradigm shift has transformed both the risk-benefit calculus and the ethical framework governing patient selection in early-phase research.

A fundamental requirement for enrolling patients in phase I trials is a physician-estimated life expectancy exceeding three months, as participation in these studies implies exposure to investigational toxicity without guaranteed therapeutic benefit. Yet retrospective analyses consistently show that up to 15% of enrolled patients die before reaching this threshold, raising both methodological and ethical concerns (2, 3). Premature death during a trial not only prevents adequate safety evaluation in those individuals but also introduces bias into dose-escalation assessments and may disproportionately affect the most vulnerable patients. Conversely, over restrictive selection criteria risk excluding patients who could meaningfully benefit from early access to novel agents, a particularly relevant concern in immunotherapy, where durable responses may occur even after apparent initial progression. The tension between protecting fragile patients from futile enrolment and preserving equitable access to potentially effective investigational therapies underscores the need for objective, validated prognostic tools that can supplement, rather than replace clinical judgment in the patient selection process.

Several prognostic scores have been developed to estimate short-term survival in early-phase trials. The Royal Marsden Hospital (RMH) score (4) — the earliest, built on albumin, lactate dehydrogenase (LDH), and number of metastatic sites — was later adapted for immunotherapy populations as the Gustave Roussy Inmune (GRIm) score (5), with the addition of neutrophil-to-lymphocyte ratio (NLR). The Lung Immune Prognostic Index (LIPI) score extended this approach to ICI-treated Non-small cell lung cancer (NSCLC) patients using derived-NLR (dNLR) (6). More recently, Phase I Prognostic Online (PIPO) score incorporated six clinical variables into a web-based decision tool (7), while Real wOrld PROgnostic (ROPRO) score offered the most complex model, drawing on 27 variables from a large real-world registry (8). Each instrument reflects a distinct approach to the same clinical problem. **Table 1** summarizes their variable composition and original survival estimates.

**Table 1:** Summary of prognostic scores used in our comparative analysis.

Score	Key variables	Risk grouping	Origin	Intended use	C-index (original study)	External validation (AUC / C-index)	Reported OS by risk group
RMH (4)	LDH, albumin, number of metastases	Low (0–1), High (2–3)	RMH, UK	General phase I trial entry	0.67	Not widely validated [9, 10]	74.1 *vs* 24.9 weeks
GRIm (5)	LDH, albumin, NLR (>6)	Low (0–1), High (2–3)	Gustave Roussy	Immunotherapy phase I trials	0.70	NSCLC: 0.68 [11–14]	17.0 *vs* 4.0 months
LIPI (6)	LDH, dNLR (>3)	Good (0), Intermediate (1), Poor (2)	NSCLC-focused	ICI response stratification	0.72	SCLC/NSCLC: 0.65–0.74 [15–17]	34 *vs* 10 *vs* 3 months
PIPO (7)	ECOG, liver metastases, LDH, albumin, dNLR, number of metastases	Good (0–2), Intermediate (3–4), Poor (5–6)	VHIO	Phase I trials with ICI & TAs	0.71	No external validation published	16.3 *vs* 6.6 *vs* 2.9 months
ROPRO (8)	27 clinical and laboratory variables ^*^	Continuous score (higher = worse prognosis) ^**^	Flatiron Health	General survival prediction	0.74	No external validation. Internal validation: 0.82 (3-month AUC)	HR 4.85 between top 10% *vs* others

*Variables include age, sex, smoking, number of metastases, hemoglobin, urea, platelets, calcium, glucose, lymphocyte/leukocyte ratio, alkaline phosphatase, total protein, alanine transferase, albumin, bilirubin, chloride, monocytes, eosinophil/leukocyte ratio, LDH, heart rate, systolic blood pressure, oxygen saturation, ECOG status, neutrophil/lymphocyte ratio, aspartate transferase/ alanine transferase ratio, body mass index and tumor stage. **ROPRO >1.18 corresponds to the top 10% of scores (90th percentile cutoff) as defined in the original study. AUC, area under the curve; dNLR, derived neutrophil-to-lymphocyte ratio; ECOG, Eastern Cooperative Oncology Group; ICI, immune checkpoint inhibitor; LDH, lactate dehydrogenase; NLR, neutrophil-to-lymphocyte ratio; NSCLC, non-small cell lung cancer; SCLC, small cell lung cancer; TAs, targeted agents; UK, United Kingdom; VHIO, Vall d’Hebron Institute of Oncology. RMH, Royal Marsden Hospital score; GRIm, Gustave Roussy Inmune score; LIPI, Lung Immune Prognostic Index score; PIPO, Phase I Prognostic Online score; ROPRO, Real wOrld PROgnostic score.

Each of these instruments was developed in distinct patient populations, using different variable sets, statistical methodologies, and clinical endpoints. Importantly, while individual scores have been evaluated in specific clinical contexts, no study to date has conducted a direct, head-to-head comparison of all five instruments within a uniform, exclusively immunotherapy-treated phase I population.

The primary aim of this study is to conduct a head-to-head comparison of the prognostic performance of these five established scores—RMH, GRIm, LIPI, PIPO, and ROPRO — in a large, real-world cohort of patients enrolled in phase I immunotherapy trials at a comprehensive cancer center. Secondary objectives include evaluating the capacity of each score to identify patients with a median overall survival below the conventional 3-month eligibility threshold, assessing tumor type-specific variability in score performance, and discussing the implications of our findings for patient selection policy and future prognostic model development.

## Methods

### Study design and population

This retrospective study included 844 patients enrolled in phase I CT involving immunotherapy at the Cancer Center Clínica Universidad de Navarra from January 2016 to March 2023. Since several of these scores were specifically developed for immunotherapy-treated populations, only patients who received at least one dose of an investigational immunotherapy agent were eligible for inclusion.

### Data collection and variables

Data collected included baseline clinical characteristics, blood biomarkers, Eastern Cooperative Oncology Group (ECOG) performance status, number and sites of metastases, and survival outcomes. These variables were used to retrospectively calculate the different prognostic scores according to its original definition:

- RMH score was computed based on albumin (<3.5 g/dL), LDH (> upper normal limit), and number of metastatic sites (>2), scored as 0–3 (4).
- GRIm score used albumin, LDH, and NLR (>6), with risk classified as low (0–1) or high (2–3) (5).
- LIPI was based on LDH and dNLR (>3), classifying patients into good (0), intermediate (1), or poor (2) prognostic groups (6).
- PIPO included six variables (ECOG, liver metastases, LDH, albumin, dNLR, and number of metastases) and stratified patients into good (0–2), intermediate (3–4), and poor (5–6) groups (7).
- ROPRO was calculated using the original 27-variable formula proposed by Becker et al. (8). ROPRO >1.18 was considered as the high-risk cutoff (top 10%).

In the working dataset, some variables had missing values, and they were imputed using mean substitution. To explore the potential impact of tumor heterogeneity on score performance and survival, we conducted a subanalysis with the five more frequent tumor types. We conducted the same analysis but based on type of treatment received.

### Statistical analysis

Categorical variables were expressed as frequencies and percentages. Quantitative variables with asymmetric distribution were described using median and interquartile range, while quantitative variables with symmetric distribution were described using mean and standard deviation. Overall survival (OS) was defined as the time from first dose of study treatment to death or last contact. C-index and area under the ROC curve (AUC) at 3 months were calculated for each score to evaluate prognostic performance. Time-dependent AUC curves were also constructed. Analyses were conducted using R-4.3.1^®^ and Python software.

## Results

**Figure 1:**
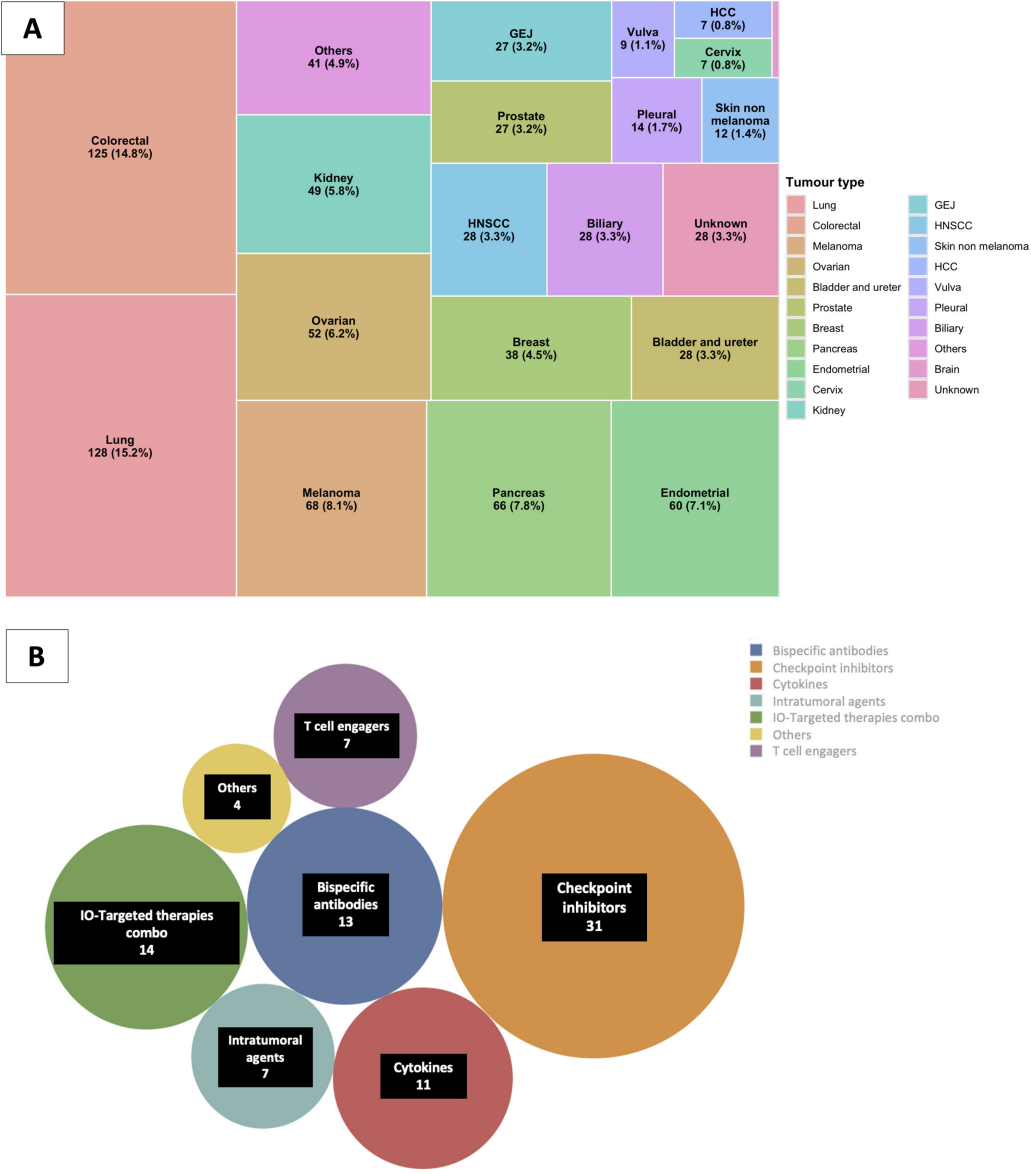
**(A)** Tumors included in our cohort of oncology patients treated in phase I clinical trials. GEJ, Gastroesophageal junction; HNSCC, Head and Neck Squamous Cell Carcinoma; HCC, Hepatocellular Carcinoma. **(B)** Types and number of clinical trials with immunotherapy. IO, Immuno-oncology.

Among the 844 oncology patients included in the study, 53.1% were male, with a median age of 59 years (**Table 2**). The most frequent tumor types were lung cancer, colorectal cancer and melanoma (**Figure 1A**). Most patients participated in trials using ICIs as monotherapy or in combination with other immunotherapeutic agents (**Figure 1B**). Data of prior received treatments was no available for the study. Median OS in the entire cohort was 9.08 months (95% CI: 8.36–9.83), from whom 20.3% (173) patients survived ≤ 3 months. Median follow-up was 9.09 months (95%CI: 3.51-13.83). The overall rate of missing values was 11.5%; these were imputed using mean substitution as described in the methods section.

**Table 2:** Clinical and demographic characteristics of oncology patients included in phase I clinical trials with immunotherapy.

	n = 844
Age (mean)	59.03 ± SD 11.42
Males (n, %) / Females (n, %)	448 (53.08%) / 396 (46.91%)
Types of tumors (n, %)	
Lung cancer	128 (15.2%)
Colorectal cancer	125 (14.8%)
Melanoma	68 (8.1%)
Ovarian cancer	52 (6.2%)
Bladder and Ureter cancer	28 (3.3%)
Prostate cancer	27 (3.2%)
Breast cancer	38 (4.5%)
Pancreas cancer	66 (7.8%)
Endometrial cancer	60 (7.1%)
Cervix cancer	7 (0.8%)
Kidney cancer	49 (5.8%)
GEJ cancer	27 (3.2%)
HNSCC cancer	28 (3.3%)
Skin non melanoma	12 (1.4%)
HCC	7 (0.8%)
Vulva cancer	9 (1.1%)
Pleural cancer	14 (1.7%)
Biliary cancer	28 (3.3%)
Others	41 (4.9%)
Brain cancer	1 (0.1%)
Unknown origin	28 (3.3%)
N° of phase I clinical trials (n)	
Check point inhibitors	31
Bispecific antibodies	13
IO-Target therapies combo	14
Cytokines	11
T-cell engagers	7
Intratumoral agents	7
Others	4
Laboratory parameters	
Hemoglobin (g/dL, median, IQR)	12.25 (11-13.5)
Platelets (109/L, median, IQR)	300 (206-338)
Leucocytes (109/L, median, IQR)	9,245 (5,522-9,022)
Neutrophiles (109/L, median, IQR)	7,010 (3,440-6,240)
LDH (UI/L, median, IQR)	259.5 (198-321)
Albumin (g/dL, median, IQR)	3.62 (3.51-3.83)
Liver metastasis (n, %)	343 (40.63%)
N° of phase I clinical trials (n)	
Median, IQR	2 (2-3)
>2 (n, %)	349 (41.35%)
Overall survival	
OS (months, median, IQR)	9.08 (95% CI: 8.36 – 9.83).

SD, Standard deviation; GEJ, Gastroesophageal junction; HNSCC, Head and Neck Squamous Cell Carcinoma; HCC, Hepatocellular Carcinoma; IQR, Interquartile range; LDH, Lactate dehydrogenase; OS, Overall survival; CI, Confidence interval.

**Figure 2:**
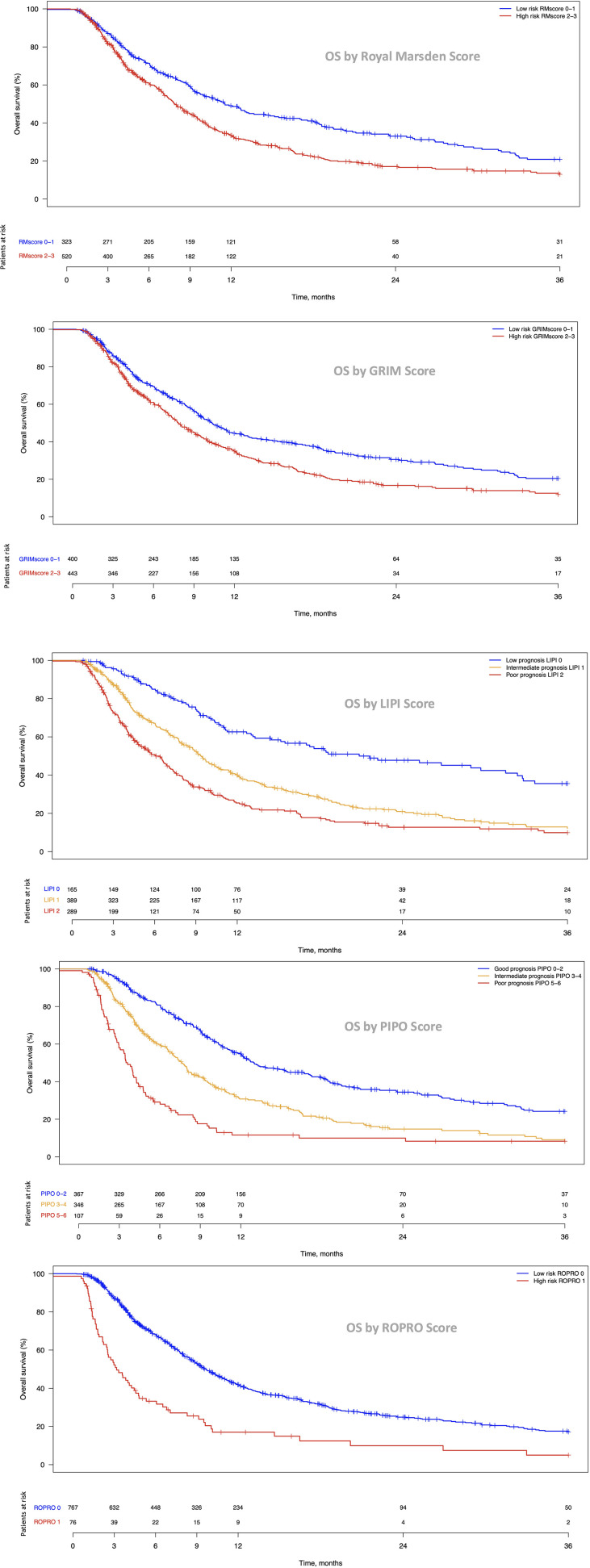
Kaplan–Meier curves of OS for different prognostic scores. Figure shows Kaplan–Meier curves of OS for each one the five scores (RMH score, GRIM score, LIPI score, PIPO score and ROPRO score). Patients are stratified according to the respective original definition. Among all scores, only patients with a high ROPRO score (score > 1.18) had a median OS approaching the clinically relevant 3-month threshold. OS, Overall survival. Analysis time: months.

Using the RMH score, 38.4% of patients were classified as low-risk (score 0–1) and 61.6% as high-risk (score 2–3). Median OS was 11.55 months (95% CI: 9.5–14.83) in the low-risk group and 7.9 months (95% CI: 7.1–8.77) in the high-risk group (HR = 1.51, 95% CI: 1.27–1.79) (**Figure 2**).

For the GRIm score, 47.4% of patients fell in the low-risk category (score 0–1) and 52.6% in the high-risk group (score 2–3). Median OS was 10.35 months (95% CI: 9.2–11.85) versus 7.84 months (95% CI: 7.08–8.95), respectively (HR = 1.40, 95% CI: 1.19–1.65) (**Figure 2**).

According to the LIPI score, 19.6% were in the good prognosis group, 46.1% in the intermediate prognosis group, and 34.4% in the poor prognosis group. Median OS was 20.86 months (95% CI: 14.48–32.36) in the good prognosis group, 9.38 months (95% CI: 8.36–10.36) in the intermediate prognosis group, and 6.39 months (95% CI: 4.85–7.10) in the poor prognosis group (HR intermediate vs. good: 1.93 [1.51–2.46]; HR poor vs. intermediate: 2.87 [2.23–3.69]) (**Figure 2**).

PIPO stratified patients into good (39.1%), intermediate (50.6%), and poor (10.3%) prognosis groups. OS was 10.86 months (95% CI: 9.78–12.53), 7.10 months (95% CI: 5.89–8.03), and 4.07 months (95% CI: 2.61–6.39), respectively. HRs were 1.58 (95% CI: 1.34–1.87) for intermediate vs. poor, and 3.07 (95% CI: 1.93–4.90) for good vs. poor (**Figure 2**).

For ROPRO, 9.1% of patients had a score >1.18 (high-risk group). These patients had a median OS of 3.31 months (95% CI: 2.48–4.55), while others had an OS of 9.65 months (95% CI: 8.79–10.66) (HR = 2.35, 95% CI: 1.82–3.05) (**Figure 2**).

**Figure 3:**
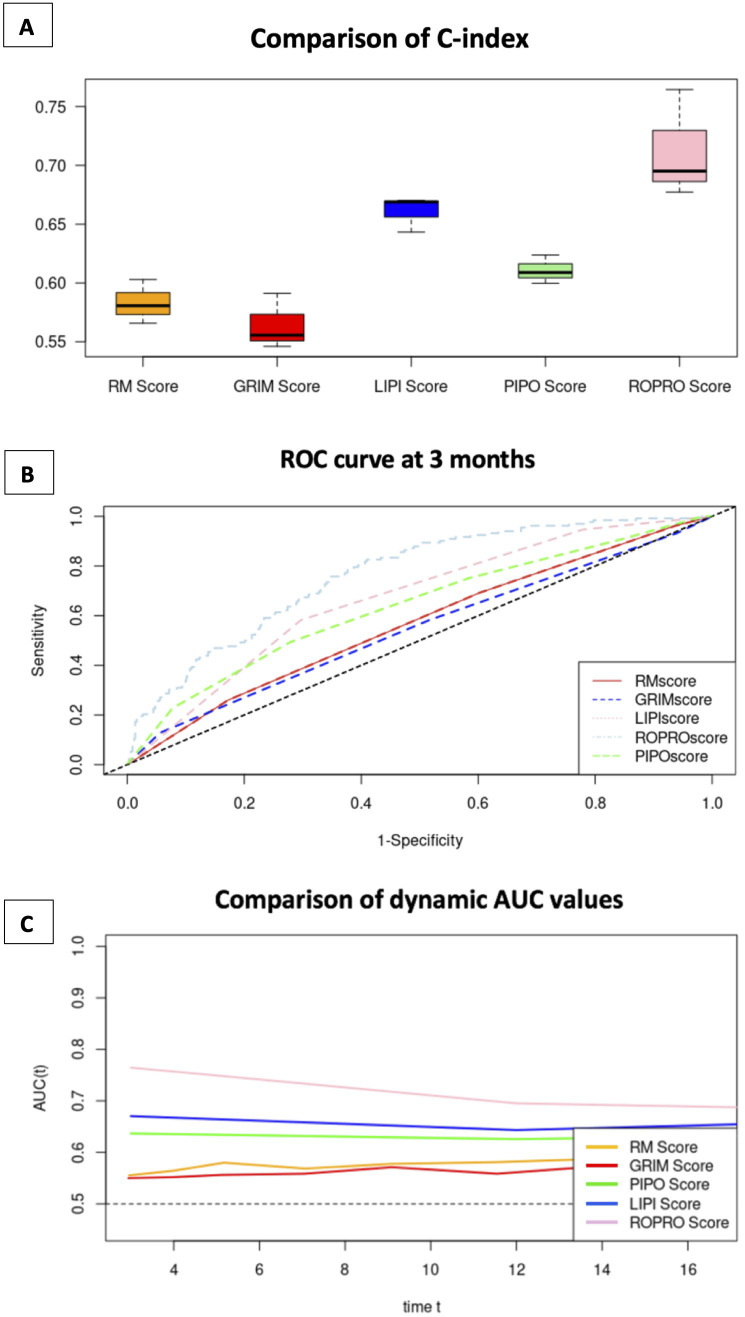
**(A)** Comparison of C-index of different prognostic scores: RMH score (0.58), GRIm score (0.55), LIPI score (0.66), PIPO score (0.63) and ROPRO score (0.69). **(B)** Area Under the Receiver Operating Characteristics (ROC) curve (AUC) at 3 months. See text for details. **(C)** Dynamic AUC values through the observation period.

The C-index values for each score were: RMH 0.58, GRIm 0.55, LIPI 0.66, PIPO 0.63, and ROPRO 0.69 (**Figure 3A**). At 3 months, the AUC was 0.56 for RMH and GRIm, 0.63 for PIPO, 0.67 for LIPI, and 0.76 for ROPRO (**Figure 3B**). Time-dependent AUC curves confirmed consistent performance, with ROPRO maintaining the highest prognostic capacity across time points (**Figure 3C**).

Additional exploratory analysis using the individual numerical values for each one of the scores rather than the predefined risk categories did not show any median OS differences. No individual score-value subgroup showed a median OS below 3 months (**Supplementary Table 1**; **Supplementary Figure 1**).

To assess the potential impact of tumor type on the performance of prognostic scores, we conducted a subgroup analysis focusing on the most prevalent malignancies in our cohort: lung cancer, colorectal cancer, melanoma, pancreas cancer and endometrial cancer. Median OS was 9.51 months (95% CI 8.65-12.55) for lung cancer, 7.18 months (95% CI 5.65-9.48) for colorectal cancer, 12.68 months (95% CI 8.49-22.21) for melanoma, 6.85 months (95% CI 4.86-8.49) for pancreas cancer and 12.19 months (95% CI 10.13-20.37) for endometrial cancer. Subsequently, all scores were recalculated for those tumor types (**Table 3**). No substantial differences in median OS were observed across any of these scenarios. Despite higher AUC for PIPO score, the median OS of the high-risk group remained above the clinically relevant 3-month threshold.

**Table 3:** Performance of prognostic scores to detect high risk patients with OS < 3 months according to the type of tumor.

Score	Tumor Type	OS (months) for classified high risk patients	AUC (3 months)
RMH	1	9.51 (95% CI 6.52-13.05)	0.67
	2	6.79 (95% CI 5.37-9.12)	0.55
	3	8.98 (95% CI 7.38-18.86)	0.62
	8	6.85 (95% CI 3.96-8.72)	0.52
	9	11.09 (95% CI 4.97-18.44)	0.83
ROPRO	1	3.17 (95% CI 1.69-NE)	0.60
	2	3.14 (95% CI 2.15-NE)	0.62
	3	8.3 (95% CI 3.61-NE)	0.52
	8	3.6 (95% CI 1.1-NE)	0.56
	9	4.55 (95% CI 1.79-NE)	0.67
LIPI	1	6.52 (95% CI 4.68-12.17)	0.69
	2	5.34 (95% CI 3.94-6.81)	0.58
	3	7.38 (95% CI 4.02-22.98)	0.75
	8	7.7 (95% CI 4.42-NE)	0.61
	9	4.61 (95% CI 2.21-NE)	0.81
GRIM	1	8.95 (95% CI 6.52-12.55)	0.68
	2	6.05 (95% CI 5.37-8.77)	0.42
	3	9.95 (95% CI 7.33-21.24)	0.59
	8	7.34 (95% CI 4.42-14.64)	0.41
	9	4.97 (95% CI 2.75-18.61)	0.87
PIPO	1	6.55 (95% CI 1.69-NE)	0.81
	2	3.83 (95% CI 3.32-6.81)	0.78
	3	4.02 (95% CI 3.61-NE)	0.77
	8	2.86 (95% CI 1.89-NE)	0.75
	9	2.74 (95% CI 1.6-NE)	0.89

Despite higher AUC for PIPO score, the median OS of the high-risk group remained above the clinically relevant 3-month threshold. 1: Lung cancer. 2: Colorectal cancer. 3: Melanoma. 8: Pancreas cancer. 9: Endometrial cancer. AUC: Area Under the Curve. OS: Overall Survival. NE: Not estimable.

Although small sample sizes limited the interpretability of several treatment-specific subgroup analyses, the same pattern was observed in treatment groups with larger patient numbers. Across these larger subgroups, high-risk score categories did not consistently correspond to a median OS below 3 months (**Supplementary Table 2**). In contrast, PIPO score performance had a greater AUC compared with LIPI and ROPRO scores, although none of the scores define a subgroup with expected survival below 3 months.

## Discussion

This study is the first to directly compare the prognostic performance of five used scores—RMH, GRIm, LIPI, PIPO, and ROPRO—in a large, real-world cohort of oncology patients treated in phase I immunotherapy trials.

The study was limited to these five scores because they cover domains repeatedly shown to matter in early-phase I trials: performance and nutritional status, plus inflammatory and disease burden. Indeed, their clinical applicability and availability of required baseline variables were important factors when chose among others. The purpose was not to identify the best prognostic score among all existing models, but to test whether representative, clinically feasible scores from different developmental contexts could identify patients with survival below the 3-month threshold relevant to phase I trial eligibility.

The study was deliberately restricted to phase I immunotherapy trials because immune-based strategies have represented a substantial proportion of available phase I studies in recent years, reflecting the increasing role of immunotherapy as a major therapeutic pillar across multiple tumor types. Identifying clinically useful prognostic tools in this specific setting is highly relevant for contemporary early drug development. Moreover, although modern phase I oncology studies increasingly incorporate expansion cohorts and preliminary efficacy endpoints, their primary objectives remain centered on safety, tolerability and dose definition. In this context, enrolling patients with an expected survival of less than 3 months may be clinically and ethically questionable. Indeed, because safety and tolerability remain central objectives of phase I trials, careful patient selection is essential to ensure that safety signals are interpretable. Patients with very limited life expectancy are at higher risk of early clinical events driven by disease progression. Identifying patients at very high risk of early death is therefore relevant not only from an ethical and patient-centered perspective, but also to preserve the scientific validity of early-phase safety assessment.

This issue is especially relevant for immunotherapy trials because, unlike rapidly cytoreductive therapies, the clinical benefit of immune-based treatments may require time to emerge including delayed responses, pseudoprogression, and hyperprogression. Therefore, patients with an expected survival below 3 months may not live long enough to experience a potential therapeutic benefit, while still being exposed to trial-related procedures, toxicity, hospital visits and the psychological burden associated with trial participation. Identifying patients at very high risk of early death is not intended to restrict access to clinical research, but rather to support a more appropriate and patient-centered selection process.

Our findings reveal modest discriminative performance across all models, with ROPRO showing the highest performance in both C-index and short-term survival prediction (AUC at 3 months). However, a key and clinically relevant observation was that all patients classified in the worst prognostic categories by any score had median overall survival greater than 3 months.

This result has profound implications: if these scores are used as rigid exclusion criteria in early-phase CT, a substantial number of patients who may benefit from participation—and have a life expectancy exceeding 3 months—could be unjustifiably excluded. As we mentioned before, this is particularly important in the context of immunotherapy, where responses can be delayed or atypical. Indeed, specific challenge in immunotherapy trials is that clinical outcomes may be influenced by patterns that are not fully captured by baseline prognostic variables. Most of the evaluated scores rely on inflammatory, nutritional and tumour-burden surrogates. While these factors are consistently associated with poor overall prognosis, they may not adequately reflect immunotherapy-specific tumor trajectories. Therefore, the limited performance of these scores in predicting very short survival should not necessarily be interpreted as a failure of their general prognostic value, but rather as a limitation of applying static baseline inflammatory and nutritional biomarkers to a dynamic treatment context such as immunotherapy.

Our analysis highlights the ethical and methodological limitations of relying solely on prognostic scores for trial eligibility decisions. While useful as complementary tools, especially for stratification or exploratory subgroup analysis, they should not replace clinical judgment nor serve as definitive cutoff tools. This aligns with recent discussions in the literature about patient-centered selection and avoiding potential harm from overexclusion (9). This also challenges the concept of “prognostic enrichment,” whereby trials attempt to selectively enroll patients with a better expected outcome to maximize the probability of detecting a treatment signal or demonstrating clinical benefit. Instead, these scores may be more valuable as stratification factors than as filters.

The relative performance of the scores in our cohort also raises questions about their generalizability. For example, the GRIm score—developed for phase I immunotherapy trials—performed worse in our dataset than reported in its original publication (5). This may reflect population differences, such as our higher proportion of colorectal cancer patients, including many with liver metastases—a group known to have poorer prognosis (10). The inclusion of tumor types with divergent biology (e.g., pancreatic vs. non-melanoma skin cancer) could also influence score performance. Although this heterogeneity reflects real-world phase I populations, our stratified approach with subanalysis supports the robustness of our findings, while acknowledging that tumor biology could eventually affect the discriminatory power of prognostic scores.

Interestingly, the median overall survival observed in our cohort appears relatively prolonged for a population enrolled in phase I trials. This may reflect an evolving landscape in early-phase oncology research, where phase I trials increasingly include patients earlier in their disease course, sometimes even as first-line therapy, rather than exclusively after multiple prior lines of treatment. This shift likely contributes to improved baseline prognosis and challenges traditional assumptions regarding expected outcomes in phase I settings.

Of note, the PIPO score had not previously been validated externally. Our data show consistent stratification across its three risk categories, but lower AUC values than originally reported (7). This underlines the need for external validation studies before wide clinical adoption.

Interestingly, simpler models like LIPI performed comparably to the more complex ROPRO. This supports the principle of parsimony (Ockham’s razor): in many settings, a score with fewer, robust variables may be as informative as one with many parameters. Simplicity does not imply inferiority. However, ROPRO’s continuous nature and integration of physiologic variables explain its superior discrimination in our analysis. Notably, even within the highest-risk ROPRO group, median OS marginally exceeded the conventional 3-month eligibility threshold, underscoring the limited utility of any single score as an exclusion criterion.

To assess whether broader grouping could have masked a subgroup with particular poor prognosis, an additional exploratory analysis was performed with the individual numerical values of each score. The lack of identification of a high-risk subgroup with median OS bellow 3 months reinforces the need of more future approaches to identify accurate tools. Analysis performed with treatment-specific subgroup supports the same conclusion although PIPO score showing a better AUC compared with LIPI and ROPRO allows us to hypothesize that each score may have a different applicability depending on the type of treatment received. Moreover, analysis performed with tumor-type subgroups provide similar thoughts. Limited number of events or censored observations influences the results obtained.

This study has several strengths, including its large sample size, exclusive focus on immunotherapy phase I trials, and head-to-head comparison of established prognostic tools. Limitations include the retrospective nature of data collection, the single-centre design which may limit generalizability, the use of mean imputation for missing values, and the absence of data on patients who were not included in trials due to physician-based selection, which could bias the estimation of score performance. Additionally, our population might not be fully representative of the broader population of cancer patients. The exclusion of other prognostic tools is also a limitation. However, the selected scores were chosen to avoid excessive redundancy between models based on similar routinely available variables and to focus on clinically interpretable tools representing distinct developmental contexts. Absence of prior treatment burden also remains a limitation and should be considered when interpreting our results.

In summary, current prognostic scores, while potentially useful for broad prognostic stratification, they offer limited guidance in identifying patients with an expected survival shorter than 3 months in the phase I immunotherapy trials setting. They should not be used to strictly exclude patients based on survival predictions. Future approaches should explore dynamic models, integration of biological markers, and artificial intelligence tools for better prognostic accuracy and patient selection.

## Data Availability

The original contributions presented in the study are included in the article/**Supplementary Material**. Further inquiries can be directed to the corresponding author.
