# Supplementary material for: Prognostic value of existing scoring systems for early mortality in phase I immunotherapy trials: a head-to-head real-world cohort study
Source: Front Oncol. 2026 Jul 20;16:1849425. doi: 10.3389/fonc.2026.1849425 (PMC13462552; doi:10.3389/fonc.2026.1849425)
Supplement: Supplementary file 1 [file DataSheet1.pdf]

Prognostic Score		n	Deaths	Median OS, months (95% CI)
**RMH Score**	RMH score 0	61	41	10.66 (8.36-18.02)
	RMH score 1	351	236	13.14 (11.85-18.6)
	RMH score 2	456	330	8.98 (8.3-10.2)
	RMH score 3	196	146	7.38 (6.02-9.38)
**GRIM Score**	GRIM score 0	60	40	9.97 (6.98-15.9)
	GRIM score 1	467	315	11.85 (10.33-15.47)
	GRIM score 2	474	348	9.05 (7.84-10.55)
	GRIM score 3	63	50	4.78 (3.96-8.46)
**LIPI Score**	LIPI score 0	235	140	20.86 (18.33-31.51)
	LIPI score 1	482	343	10.14 (9.2-11.48)
	LIPI score 2	347	270	6.69 (5.75-7.61)
**PIPO Score**	PIPO score 0	57	28	43.01 (32.36-51.34)
	PIPO score 1	173	110	18.17 (15.34-27.5)
	PIPO score 2	250	184	10.4 (9.54-12.5)
	PIPO score 3	231	160	9.32 (8.07-10.95)
	PIPO score 4	194	145	7.38 (6.05-8.36)
	PIPO score 5	106	90	4.02 (3.43-4.8)
	PIPO score 6	23	17	3.08 (1.69-6.81)

Supp. Table 1. Median OS of individual score-value subgroups for all scores. Individual numerical value of each score was used to calculate the prognosis instead of the predefined risk categories.

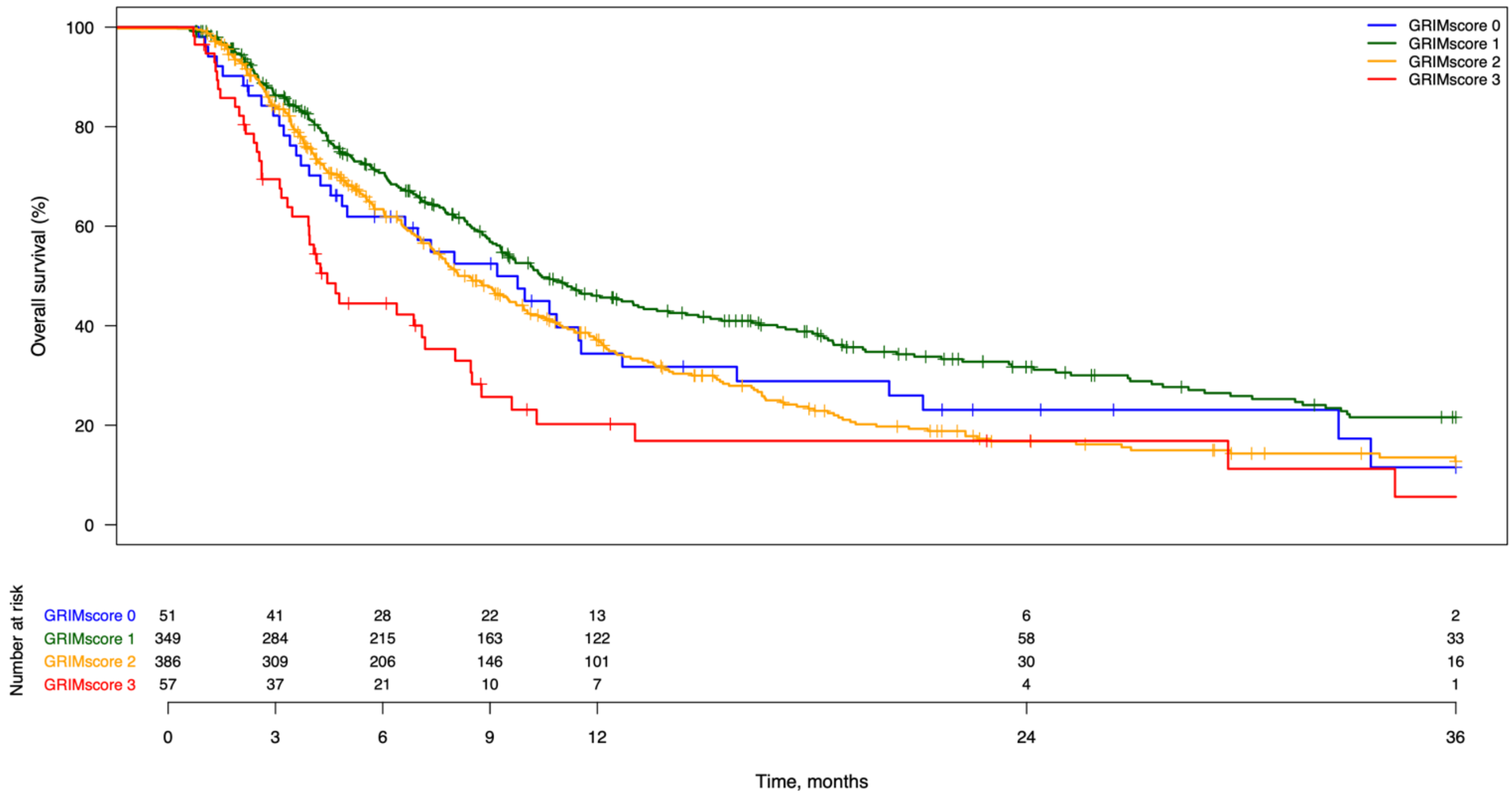

GRIMscore 0
GRIMscore 1
GRIMscore 2
GRIMscore 3
Overall survival (%)
100
80
60
40
20
0
Number at risk
GRIMscore 0 51 41 28 22 13 6 2
GRIMscore 1 349 284 215 163 122 58 33
GRIMscore 2 386 309 206 146 101 30 16
GRIMscore 3 57 37 21 10 7 4 1
0 3 6 9 12 24 36
Time, months

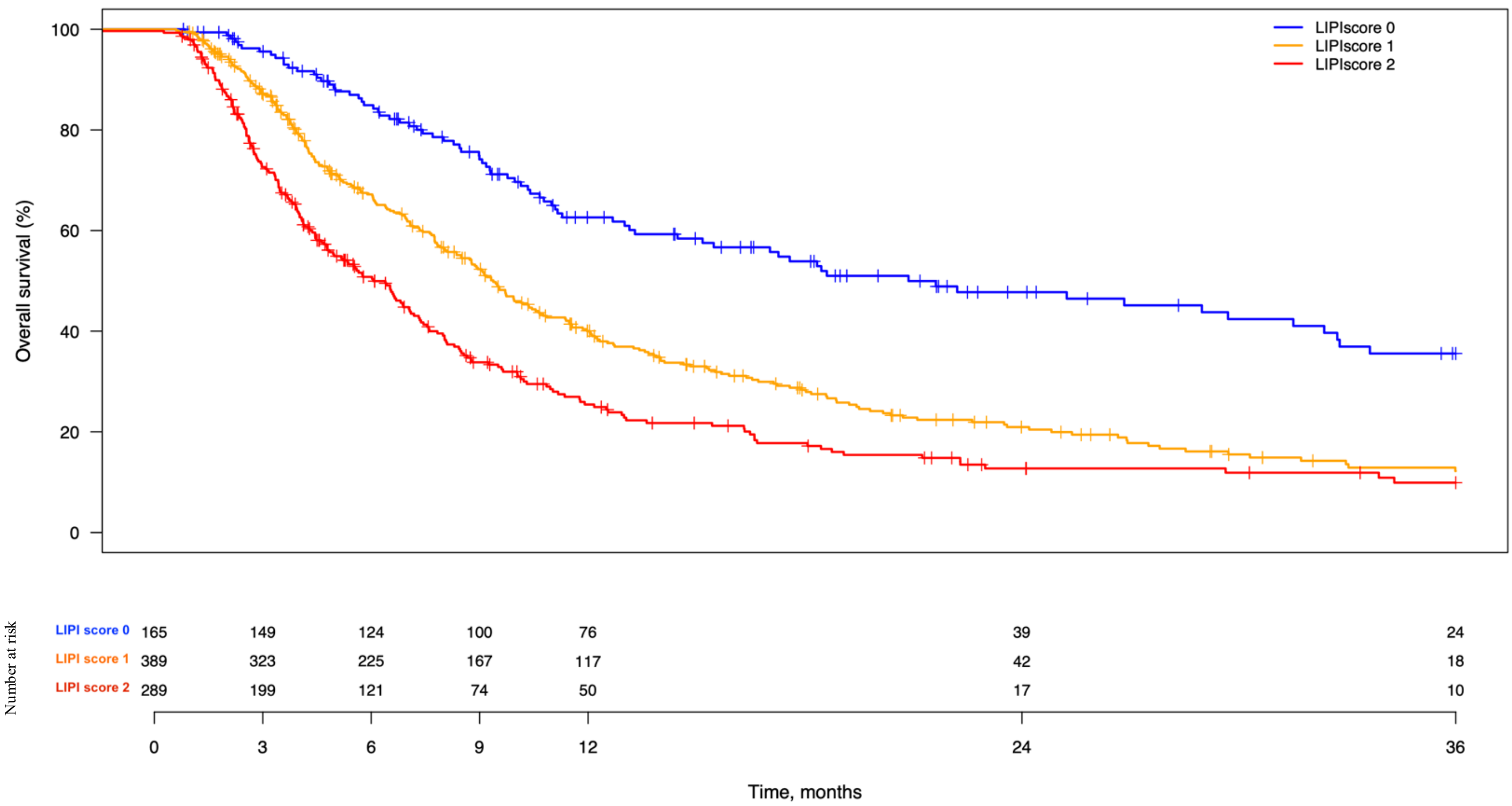

LIPIscore 0
LIPIscore 1
LIPIscore 2
Overall survival (%)
100
80
60
40
20
0
Number at risk
LIPI score 0 165 149 124 100 76 39 24
LIPI score 1 389 323 225 167 117 42 18
LIPI score 2 289 199 121 74 50 17 10
0 3 6 9 12 24 36
Time, months

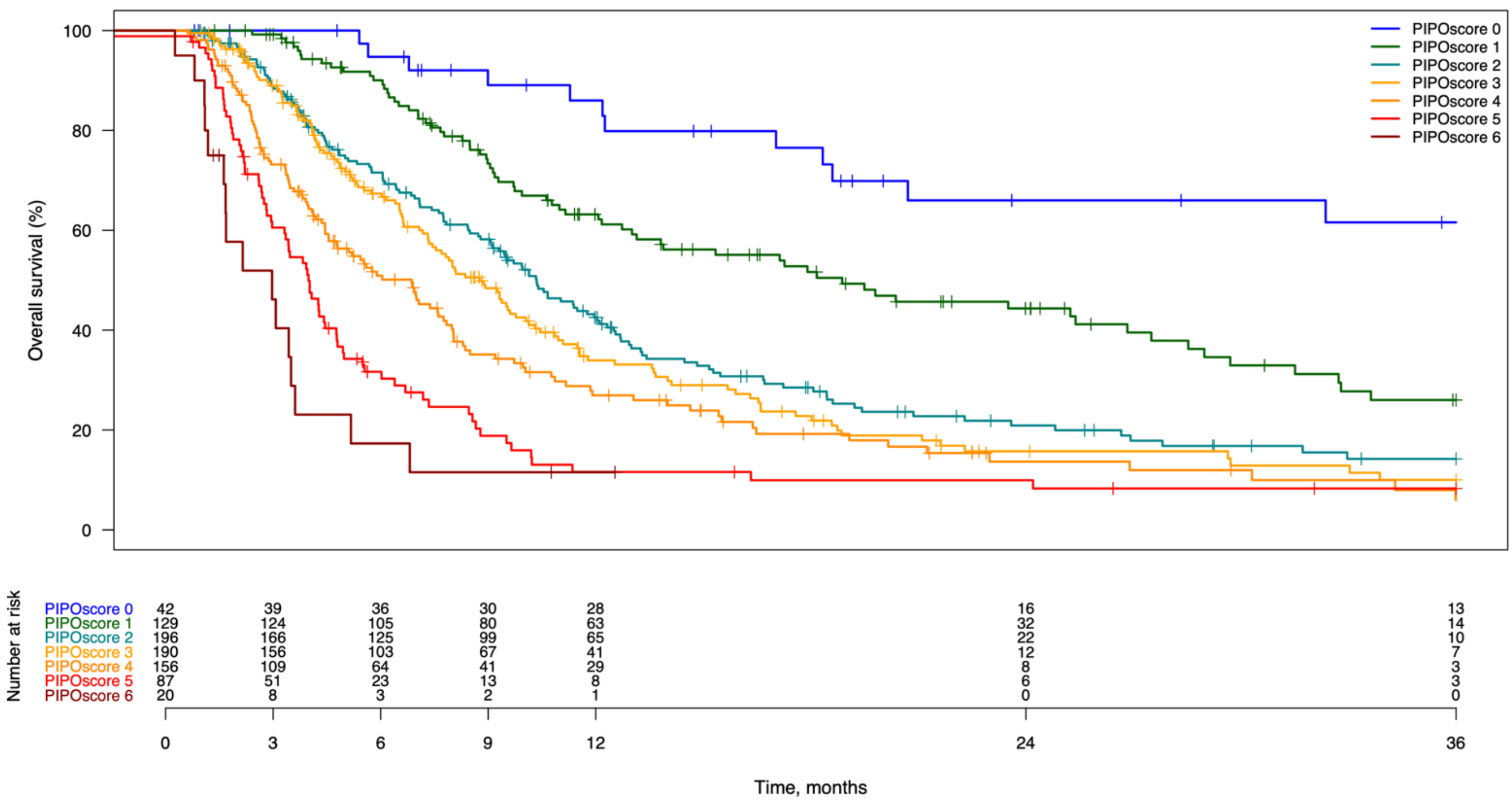

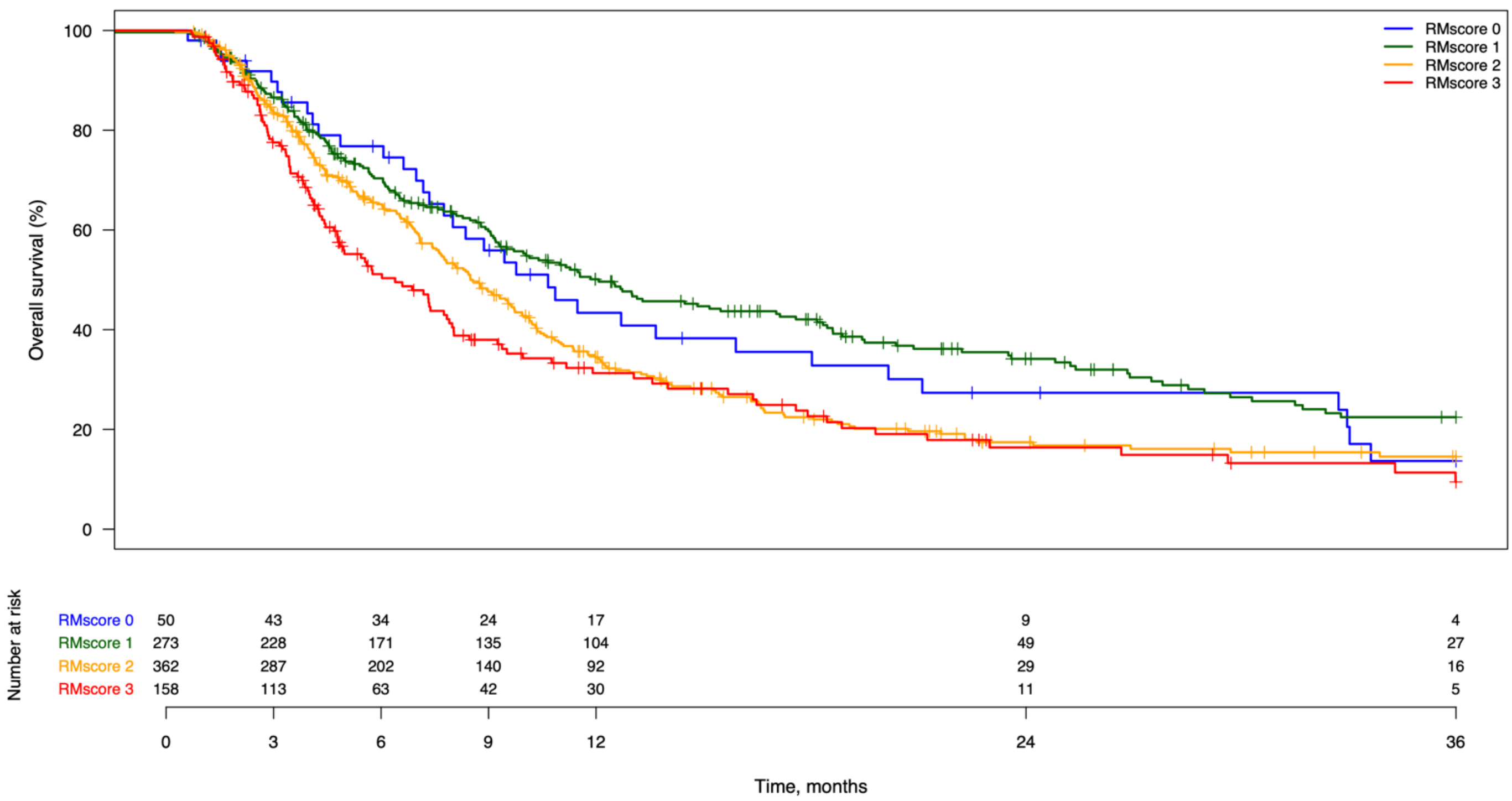

Supp. Figure 1. Kaplan Meier curves of individual score-value subgroups for RMH, GRIM, LIPI and PIPO scores. Individual value for each score did not add prognostic capacity compared with the predefined risk categories.

SCORE	Treatment subgroup	OS (months) for classified high risk patients	AUC (3 months)
**RMH**	1	7,83 (IC95% 6.94-11.95)	0.62
	2	5.55 (IC95% 3.94-7.98)	0.56
	3	8.72 (IC95% 7.42-10.36)	0.51
**ROPRO**	1	2.15 (IC95% 1.60-NE)	0.65
	2	2.21 (IC95% 1.69-4.76)	0.64
	3	3.58 (IC95% 2.48-9.13)	0.60
**LIPI**	1	6.39 (IC95% 4.61-8.65)	0.63
	2	3.94 (IC95% 3.35-7.04)	0.65
	3	6.88 (IC95% 5.65-9.25)	0.67
**GRIM**	1	7.75 (IC95% 6.39-12.25)	0.59
	2	5.55 (IC95% 3.83-7.98)	0.58
	3	8.72 (IC95% 7.42-10.36)	0.49
**PIPO**	1	3.46 (IC95% 2.18-6.39)	0.79
	2	3.14 (IC95% 2.10-4.76)	0.79
	3	4.25 (IC95% 2.94-7.34)	0.72

Supp. Table 2. **Performance of prognostic scores to detect high risk patients with OS < 3 months according to the treatment received.** PIPO score performance had a greater prognostic capacity compared with LIPI and ROPRO scores, although none of the scores define a subgroup with expected survival below 3 months. 1: Immune check point inhibitor. 2: Bispecific antibodies. 3: Targeted therapies combo. AUC: Area Under the Curve. OS: Overall Survival. NE: Not estimable.
